# The *tailless* Ortholog *nhr-67* Regulates Patterning of Gene Expression and Morphogenesis in the C. elegans Vulva

**DOI:** 10.1371/journal.pgen.0030069

**Published:** 2007-04-27

**Authors:** Jolene S Fernandes, Paul W Sternberg

**Affiliations:** 1 Division of Biology, California Institute of Technology, Pasadena, California, United States of America; 2 Howard Hughes Medical Institute, California Institute of Technology, Pasadena, California, United States of America; Huntsman Cancer Institute, United States of America

## Abstract

Regulation of spatio-temporal gene expression in diverse cell and tissue types is a critical aspect of development. Progression through Caenorhabditis elegans vulval development leads to the generation of seven distinct vulval cell types (vulA, vulB1, vulB2, vulC, vulD, vulE, and vulF), each with its own unique gene expression profile. The mechanisms that establish the precise spatial patterning of these mature cell types are largely unknown. Dissection of the gene regulatory networks involved in vulval patterning and differentiation would help us understand how cells generate a spatially defined pattern of cell fates during organogenesis. We disrupted the activity of 508 transcription factors via RNAi and assayed the expression of *ceh-2,* a marker for vulB fate during the L4 stage. From this screen, we identified the *tailless* ortholog *nhr-67* as a novel regulator of gene expression in multiple vulval cell types. We find that one way in which *nhr-67* maintains cell identity is by restricting inappropriate cell fusion events in specific vulval cells, namely vulE and vulF. *nhr-67* exhibits a dynamic expression pattern in the vulval cells and interacts with three other transcriptional regulators *cog-1* (Nkx6.1/6.2), *lin-11* (LIM), and *egl-38* (Pax2/5/8) to generate the composite expression patterns of their downstream targets. We provide evidence that *egl-38* regulates gene expression in vulB1, vulC, vulD, vulE, as well as vulF cells. We demonstrate that the pairwise interactions between these regulatory genes are complex and vary among the seven cell types. We also discovered a striking regulatory circuit that affects a subset of the vulval lineages: *cog-1* and *nhr-67* inhibit both one another and themselves. We postulate that the differential levels and combinatorial patterns of *lin-11, cog-1,* and *nhr-67* expression are a part of a regulatory code for the mature vulval cell types.

## Author Summary

During development, in which the single-celled egg generates a whole organism, cells become different from each other and form patterns of types of cells. It is these spatially defined fate patterns that underlie the formation of complex organs. Regulatory molecules called transcription factors influence the fate patterns that cells adopt. Understanding the role of these transcription factors and their interactions with other genes could tell us how cells establish a certain pattern of cell fates. This study focuses on studying how the seven cell types of the Caenorhabditis elegans vulva arise. This organ is one of the most intensively studied, and while the signaling network that initiates vulval development and sets the gross pattern of cell differentiation is well understood, the network of transcription factors that specifies the final cell fates is not understood. Here, we identify *nhr-67,* a new transcription factor that regulates patterning of cell fates in this organ. Transcription factors do not necessarily act alone, and we explore how NHR-67 works with three other regulatory factors (each with human homologs) to specify the different properties of the vulval cells. We also demonstrate that the interconnections of these transcription factors differ between these seven diverse cell types, which may partially account for how these cells acquire a certain pattern of cell fates.

## Introduction

Complex gene regulatory networks operating in diverse cell types and tissues are crucial for development. Diverse intercellular signals and transcription factor networks control gene expression within individual cell types, acting on *cis*-regulatory modules of target genes [1]. Understanding such regulation first requires documenting all the regulatory inputs and outputs from each gene [2]. This information allows circuit diagrams to be constructed that provide a global perspective on how diverse cell types acquire their identity. Gene regulatory networks have been well studied in a wide range of biological model systems such as endomesoderm specification in the sea urchin embryo [3], dorso-ventral patterning in the *Drosophila* embryo [4], and mesoderm specification in *Xenopus* [5]. The common themes that might emerge from these studies would advance our understanding of organogenesis in vertebrates.

The Caenorhabditis elegans vulva is postembryonically derived from six vulval precursor cells P3.p–P8.p. The central three vulval precursor cells P5.p–P7.p are induced to adopt 1° (primary) and 2° (secondary) vulval fates via epidermal growth factor (EGF) and Notch signaling, whereas the remaining precursors fuse with the hypodermal syncytium hyp7 [6]. The vulva is composed of seven distinct cell types, each with its own set of expressed genes and morphogenetic migrations [7–9]. The P6.p 1° lineages generate the vulE and vulF cells, while the P5.p and P7.p 2° lineages generate the vulA, vulB1, vulB2, vulC, and vulD cells. The signals that induce 1° versus 2° fates in the primordial vulval precursor cells are known. However, the processes that govern patterning and differentiation of the mature vulval cell types are largely unknown [6]. Both Ras and Wnt pathways are required for the precise spatial patterning of the 1° vulE and vulF cells [10], and both Wnt/Ryk and Wnt/Frizzled signaling pathways are necessary for patterning the P7.p 2° vulA–vulD cells [11–13].

Genes expressed in the mature vulval cell types include some with known functions and many others without known physiological roles. *lin-3* (EGF) is expressed in vulF and is required to signal from vulF to uterine uv1 cells [14,15]. *egl-17* encodes a fibroblast growth factor (FGF)-like protein that is required for migration of the sex myoblasts to their precise final positions [16,17]. *egl-17* is initially expressed in the 1° vulval lineages and is shut off during the L4 stage. Expression in vulC and vulD is observed during early L4 and persists throughout adulthood. The vulval expression correlates with the sites of muscle attachment. *egl-26* encodes a novel protein that contains an H box/NC domain and is expressed in vulB1, vulB2, vulD, and vulE cells [18,19]. *zmp-1* encodes a zinc metalloprotease and is expressed in vulD and vulE during the L4 stage and in vulA in adults [9]. *ceh-2* encodes a homeodomain protein that is related to *Drosophila empty spiracles* and is expressed in vulB1 and vulB2 cells during the L4 stage and in vulC upon entry into L4 lethargus [9,20]. *pax-2* is a recent gene duplication of the PAX2/5/8 protein EGL*-*38 [21] and is expressed exclusively in the vulD cells. *zmp-1, ceh-2, egl-26,* and *pax-2* have no known function in the vulva.

Transcription factor networks in individual vulval cell types somehow generate a spatially precise pattern of cell fates [19]. Several transcription factors that regulate gene expression in the diverse vulval cell types have already been described [19,22–24]. *lin-11,* a LIM homeobox transcription factor, regulates gene expression in all seven vulval cell types [25,26]. The Nkx6.1/Nkx6.2 homeodomain gene, *cog-1,* regulates gene expression in vulB, vulC, vulD, vulE, and vulF cells [19,27]. In contrast, *egl-38* encodes a PAX2/5/8 protein that appears to be the only known example of a vulval cell type–specific regulatory factor; it promotes expression of certain target genes and restricts expression of other targets exclusively in vulF cells [14,19,28]. Additional regulatory factors need to be identified to elucidate the precise spatial patterning of the mature vulval cell types.

Here, we identify *nhr-67* as a component of the gene regulatory networks underlying vulval patterning and differentiation. *nhr-67* is required for the accurate patterning of gene expression and regulation of cell fusion in several vulval cell types and is dynamically expressed in the vulva. *nhr-67* interacts genetically with *cog-1, egl-38,* and *lin-11* to produce the complex expression patterns of their downstream targets. We demonstrate that the pairwise interactions between these four regulatory genes vary among the diverse vulval cell types. These results indicate that *nhr-67, cog-1, lin-11,* and *egl-38* form a part of a genetic network that generates different patterns of gene expression in each of the seven cell types.

## Results

### *nhr-67* Regulates Gene Expression in Multiple Vulval Cell Types

**Figure 1:**
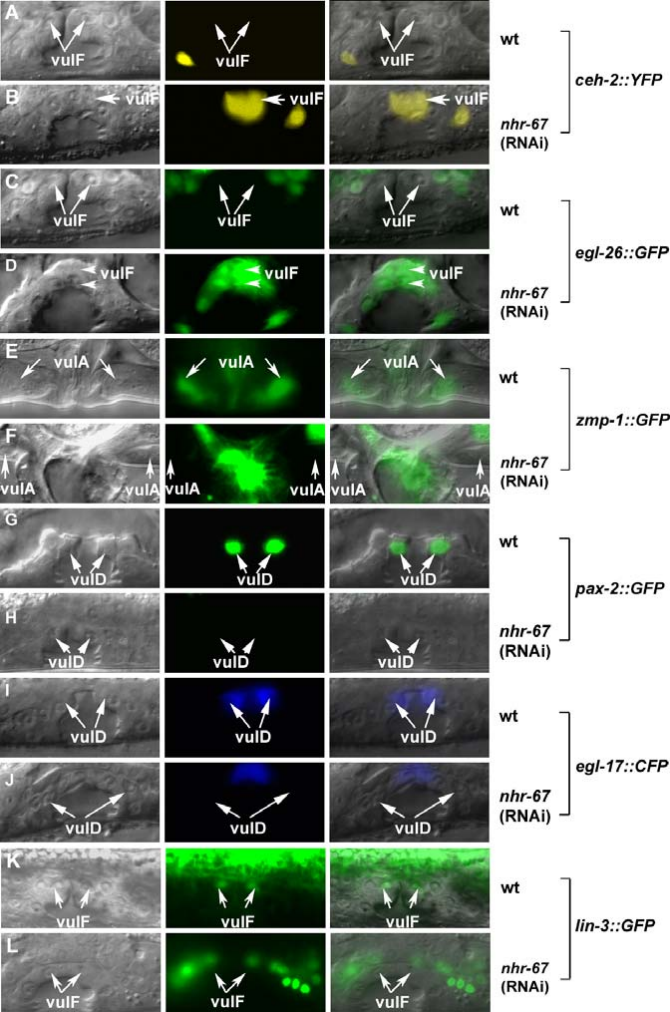
*nhr-67* Is Required for Proper Gene Expression in Multiple Vulval Cell Types Lateral images of the developing vulva during the L4 (A–D and G–L) and the adult stage (E and F). (A–L) Nomarski (left), fluorescence (center), and overlaid (right). Expression of vulval cell fate markers in wild-type (A, C, E, G, I, and K) and *nhr-67* RNAi–treated animals (B, D, F, H, J, and L). In *nhr-67* RNAi–treated animals, the vulval morphology is abnormal compared to wild-type; namely, the migration of vulF cells is defective. (A) In wild-type animals, *syIs55 [ceh-2::YFP]* expression is off in the 1° vulF cells (arrows). (B) *syIs55* animals treated with *nhr-67* RNAi show ectopic *ceh-2* expression in the 1° vulF cells (arrow). (C) *kuIs36 [egl-26::GFP]* expression is completely absent in the vulF cells (arrows). (D) *nhr-67* RNAi results in misexpression of *egl-26* in the vulF lineages (arrowheads). (E) Wild-type *zmp-1::GFP (syIs49)* expression is observed in the vulA cells (arrows). (F) In contrast, *nhr-67* RNAi abolishes the vulA-specific expression (arrows) of *zmp-1.* (G) *guEx64 [pax-2::GFP]* is expressed exclusively in vulD cells (arrows) in wild-type animals. (H) *pax-2* expression in vulD is abolished in an *nhr-67* (RNAi) background (arrows). (I) Wild-type *egl-17::GFP (syIs59)* expression is observed in the vulD cells (arrows). (J) *nhr-67* RNAi results in the loss of *egl-17* expression in the vulD cells (arrows). (K) In wild-type animals, *lin-3::GFP (syIs107)* is expressed solely in vulF cells (arrows). (L) *lin-3* expression in vulF cells is eliminated when treated with *nhr-67* RNAi (arrows).

An RNA interference (RNAi) screen of 508 known and putative transcription factors encoded in the C. elegans genome (see Table S1) was conducted in a *ceh-2::YFP* reporter background. At the time we performed the screen, this was the best available set. *ceh-2* encodes a homeodomain protein orthologous to *Drosophila* Empty Spiracles (EMS) and vertebrate EMX1 and EMX2 and serves as a readout for vulB fate during the L4 stage [20]. Modifiers of *ceh-2* expression are good candidates for genes involved in patterning and/or differentiation of 2° vulval descendents. From this screen, we identified *nhr-67* as a gene necessary for negative regulation of *ceh-2* expression in the 1° vulE and vulF cells (Figure 1A–1B). Reciprocal BLAST searches indicate that *nhr-67* encodes an ortholog of the *tailless* hormone receptor, which consists of an N-terminal transactivation domain, a centrally positioned DNA-binding domain, and a C-terminal ligand-binding domain. The only other positive was the GATA-type transcription factor *egl-18,* which was previously shown to be involved in vulval development [29–31]. Other genes that should have been positive in the screen *(lin-11* and *cog-1)* were not isolated from the RNAi screen, thus indicating a high false-negative rate. Analysis of the *nhr-67* deletion allele *ok631* revealed severe defects in early larval development (L1 lethality and/or arrest). In order to bypass this early larval arrest phenotype, we resorted to feeding young L1 larvae with *nhr-67* RNAi and assayed for defects in vulval gene expression. *nhr-67* was also found to be required for negative regulation of two additional L4-specific markers: *egl-26* (wild-type expression in vulB, vulD, and vulE cells) (Figure 1C–1D) and *egl-17* (wild-type expression in vulC and vulD cells) in the vulF cells. Thus, *nhr-67* activity is necessary for the negative regulation of expression of several 2° lineage-specific genes in the 1°-derived vulval cells during the L4 stage. Consistent with previous reports, *nhr-67* RNAi results in a highly penetrant protruding vulva (Pvl) and egg-laying (Egl) defective phenotype [32] (Figure S1). However, other transcription factors exhibiting a Pvl RNAi phenotype, such as *fos-1, egl-43,* and *unc-62,* have normal vulval gene expression (unpublished data).

In addition to its negative regulatory role, we also found that *nhr-67* is necessary for promoting expression of specific genes. For example, *nhr-67* is necessary for *zmp-1* expression in vulA during the adult stage (Figure 1E–1F). *nhr-67* is also required for vulD-specific expression of *pax-2* and *egl-17* during the L4 stage (Figure 1G–1J). These examples show that *nhr-67* positively regulates gene expression in the secondary vulA and vulD cells. *nhr-67* is also required for positively regulating gene expression in the 1° vulval cells, namely vulF-specific expression of *lin-3,* an EGF-like protein (Figure 1K and 1L). Therefore, *nhr-67* regulates gene expression in at least four of the seven vulval cell types.

In the L3 stage, the early 1° and 2° vulval cell fates can be distinguished by the patterns of cell division of their descendents. The 1° fated cell typically gives rise to four granddaughters that divide transversely (left-right axes); whereas a subset of the granddaughters derived from a 2° cell divide longitudinally (anterior-posterior axes). To determine if *nhr-67*-dependent alterations in gene expression are a consequence of fate transformations in the early 1° and 2° vulval lineages, we monitored the pattern of the vulval cell divisions in an *nhr-67* RNAi background. In the absence of *nhr-67,* the vulval cell lineages appear wild-type in terms of both cell number and orientation of cell division (unpublished data). Thus, the perturbations in gene expression caused by reduced *nhr-67* function are not the result of gross abnormalities in the early vulval cell lineages.

### *nhr-67* Prevents Inappropriate Fusion Events between the 1° Vulval Cells

**Figure 2:**
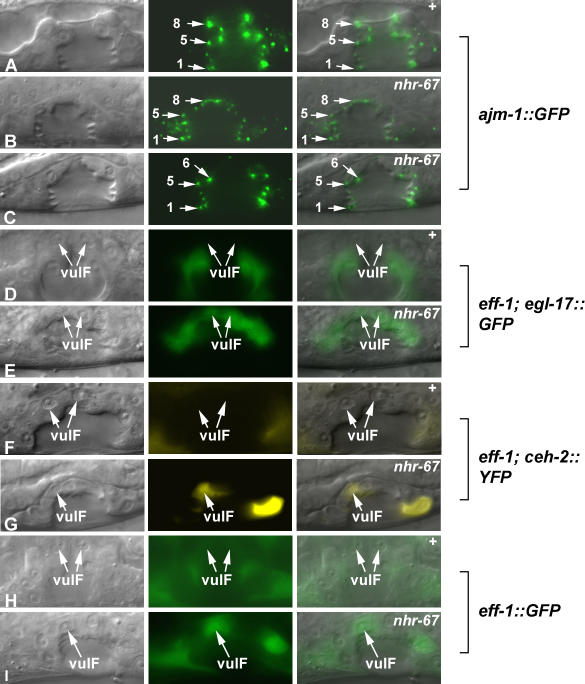
*nhr-67* Prevents Inappropriate Fusion Events between the 1° Vulval Cells (A–I) Nomarski (left), fluorescence (center), and overlaid (right). The adherens junction marker *ajm-1::GFP* is used to visualize the cell number and architecture of the vulval toroids in wild-type (A) and *nhr-67* RNAi–treated animals (B and C). When observing a mid-sagittal optical section of L4 hermaphrodites, *ajm-1::GFP* appears as dots between the vulval cells. Loss of adherens junction expression signifies a reduction in the cell number due to a cell fusion defect. (A) In wild-type animals, the eight dots on either side correspond to the seven distinct vulval cell types (arrows). The overall vulval morphology of *nhr-67* RNAi–treated animals appears abnormal compared to wild-type. (B) In some cases, the number of adherens junctions is normal in *nhr-67* RNAi–treated animals (arrows). (C) Reduction of *nhr-67* sometimes results in the loss of dots at the top of the vulval invagination (which indicates an inappropriate fusion event between the vulE and vulF cells) (arrows). However, the altered gene expression observed in an *nhr-67* RNAi background does not appear to be dependent on cell fusion defects. (D) In the absence of *eff-1*-mediated fusion, *ayIs4 [egl-17::GFP]* expression is completely absent in the vulF cells (arrows). (E) In contrast, depletion of *nhr-67* activity in an *eff-1* mutant background is sufficient to cause derepression of *egl-17* in the 1° vulF cells (arrows). (F) In *eff-1* mutants, *ceh-2* expression is absent in vulF cells (arrows). (G) Reduction of *nhr-67* activity in an *eff-1* mutant background results in ectopic *ceh-2* expression in vulF cells (arrow). (H) In wild-type animals, *eff-1::GFP* is not expressed in vulF cells (arrows). (I) *eff-1* levels are elevated in vulF cells when *nhr-67* activity is compromised (arrow).

During the L4 stage, the seven vulval cell types invaginate cooperatively to assume a characteristic morphology. The similar cell types subsequently fuse, generating toroid rings that line the vulval cavity [8]. We wanted to ascertain if the observed cell fate transformations in *nhr-67*(RNAi) animals were possibly due to improper fusion events between the wrong cell types. Cell fusion defects can be assayed using *ajm-1::GFP* (an adherens junction marker) to visualize the cell number and architecture of the vulval toroids. When observing the mid-sagittal plane of wild-type animals, *ajm-1::GFP* appears as dots between cells. The eight dots on either side correspond to the seven distinct vulval cell types (Figure 2A). Most *nhr-67* RNAi–treated animals do not exhibit dramatic defects in cell fusion (Figure 2B). The 2° vulval lineage–derived cells (vulA, vulB1, vulB2, vulC, and vulD) consistently generate mature toroids. However, inappropriate fusion often occurs (65%, *n* = 17) between the presumptive vulE and vulF cells (indicated by the missing dots at the top of the vulval invagination) (Figure 2C). Since *nhr-67* regulates gene expression in vulval cells other than vulE and vulF, improper cell fusion events cannot fully account for all its altered gene expression patterns.

We then wanted to determine if the altered gene expression occurring in the 1° vulval cells was dependent on these improper fusion events. We attempted to address this question using two approaches: (a) by analyzing the effect of *nhr-67* RNAi on the expression of *egl-17* and *ceh-2* transgenes in an *eff-1(hy21)* background, and (b) by monitoring the vulval expression levels of *eff-1* in animals with reduced *nhr-67* activity. *eff-1* is a type I membrane protein necessary for cell fusion [33]. Disruption of *nhr-67* function in an *eff-1*-deficient background is still sufficient to cause upregulation of both *egl-17* (Figure 2D and 2E) and *ceh-2* (Figure 2F and 2G) in the 1° vulval cells. Thus, the *nhr-67-*dependent alterations in gene expression are not dependent on *eff-1*-mediated cell fusion. We also observed that *eff-1* levels (strong expression in vulA and vulC cells, weak expression in vulF cells) are highly elevated in vulD and vulF cells when *nhr-67* gene activity is compromised (Figure 2H and 2I). However, we also note that *eff-1* is not sufficient to rescue the vulE-vulF fusion defects observed in *nhr-67* (RNAi) background (unpublished data). One possibility is that *eff-1(hy27)* is a temperature-sensitive allele that fails to completely eliminate cell fusion. Another possibility is that in addition to *eff-1, nhr-67* negatively regulates other target genes that mediate cell fusion.

### *nhr-67* Is Dynamically Expressed in Multiple Vulval Cells

**Figure 3:**
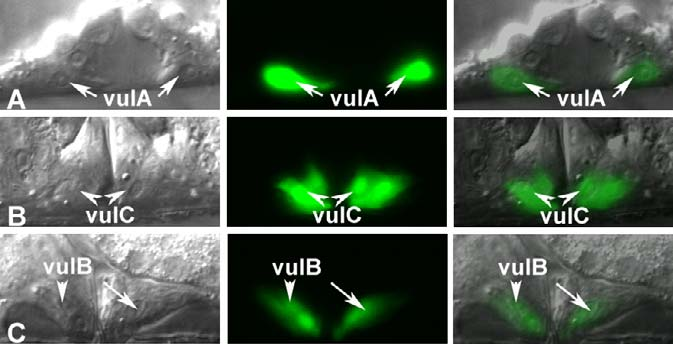
*nhr-67* Is Dynamically Expressed in Multiple Vulval Cell Types (A–C) Nomarski (left), fluorescence (center), and overlaid (right). All animals displayed carry the *syEx716* transgene in their background. (A) *nhr-67* is robustly expressed in the vulA cells (arrows) during the L4 stage. (B) vulC expression (arrowheads) is visible upon entry into L4 lethargus. (C) High levels of *nhr-67* expression in vulB1 and vulB2 cells (arrowhead and arrow) are detectable in young adults.

**Figure 4:**
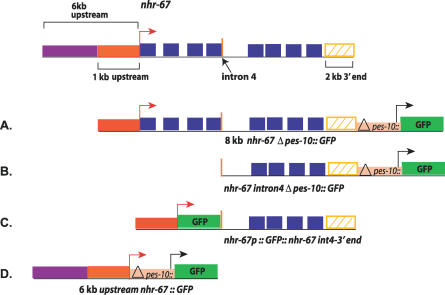
The Regulatory Element(s) Driving the Vulval Expression of *nhr-67* Is Present in the Region That Spans from the Fourth Intron to the 3′ Noncoding Region Several transcriptional reporter constructs containing the *nhr-67* coding exons (blue rectangles), introns (black lines), and the 3′ noncoding region (yellow hatched rectangle) were generated. The red arrow indicates the presumptive promoter of *nhr-67* and the black arrow is proximal to the minimal *Δpes-10 promoter.* The yellow rectangle includes 2 kb of the 3′ noncoding region. The orange vertical bar indicates the junction between the fourth exon and fourth intron. Construct (A) consists of 1 kb upstream promoter sequence (red rectangle), the entire coding region (blue rectangles) and introns (black lines), and 2 kb of the 3′ noncoding region (yellow hatched rectangle) attached to minimal *Δpes-10::GFP.* Construct (B) spans from the fourth intron (gene sequence downstream of the orange vertical bar) to the 3′ noncoding region (yellow rectangle) fused to minimal *Δpes-10::GFP.* Construct (C) is an *nhr-67::GFP* transcriptional reporter driven by 1 kb of the native promoter region (red rectangle) and contains regulatory sequences 3′ of the fourth exon (sequences downstream of the orange vertical bar) and the native 3′ noncoding region (yellow rectangle). Construct (D) contains 6-kb sequence upstream of the predicted first ATG of *nhr-67* (purple and red rectangles) appended to minimal *Δpes-10::GFP.*

Previous work reported that an *nhr-67* construct containing 6 kb of the promoter region directs expression in several head neurons [34]. We generated several additional transcriptional reporter constructs that tested the entire *nhr-67* coding region, introns and the 3′ noncoding region for enhancer activity using the *Δpes-10* basal promoter [35]. An 8-kb fragment that consisted of 1-kb 5′ sequence, the entire coding region and introns, and 2 kb of the 3′ noncoding region yielded expression in the vulva, the hyp7 epidermal syncytium, late stage embryos, and the male tail (Figures 3 and 4A). This *nhr-67* construct exhibits a dynamic expression pattern in the vulval cells. During the late L4 stage, *nhr-67* is first observed in vulA cells (Figure 3A) (and occasionally in vulB1), and this expression is maintained throughout adulthood. Expression in vulC is only seen upon entry into L4 lethargus and persists in adults (Figure 3B). Strong vulB1 and vulB2 expression (and occasional vulD expression) is observed only in young adults (Figure 3C). A 4.5-kb reporter construct that spans from the fourth intron to the 3′ noncoding region is sufficient to drive expression in the same tissues as seen with the 8-kb fragment (Figure 4B). No expression is seen in the vulC, vulD, vulE, and vulF cells during the L4 stage unless *nhr-67* or *cog-1* activity is eliminated (see below). Thus, the *cis*-elements driving the vulval expression of *nhr-67* appear to be located in the region spanning the fourth intron to the 3′ noncoding region. We then wanted to confirm if these regulatory elements were capable of interacting with the endogenous promoter of *nhr-67* in order to promote its transcription in the vulva. To test this, we generated an *nhr-67* transcriptional reporter driven by 1 kb of its native promoter and containing regulatory sequences downstream of the fourth exon in their normal context. The *nhr-67* transcriptional construct containing the endogenous promoter recapitulated the vulval and embryonic expression pattern observed with the *nhr-67::Δpes-10* constructs (Figure 4C). We also examined whether the upstream regulatory sequences of *nhr-67* interact with the downstream regulatory elements to influence its vulval expression. This test was accomplished by coinjecting a transcriptional green fluorescent protein (GFP) construct that contains a 6-kb upstream sequence of *nhr-67* (Figure 4D) with the 8-kb *nhr-67::Δpes-10* construct (Figure 4A) described above. We find that in the presence of the 6-kb promoter region, the vulval expression is identical to that of the 8-kb *nhr-67::Δpes-10* constructs. Besides the previously reported expression in head neurons, we observed expression in the anchor cell (AC) (during mid–late L3 stage) in hermaphrodites and the linker cell in males (Figure S2A and S2B).

### Regulation of *egl-17* and *ceh-2* Expression in the 1° Vulval Cells

We attempted to understand the *trans*-regulation of vulval expression in the diverse cell types by analyzing the regulation of two target genes in detail: *egl-17* and *ceh-2.* To dissect the *trans*-regulation of these target genes, we constructed various double and triple mutant/RNAi combinations and assayed for alterations in gene expression in the 1° vulval cells.

**Figure 5:**
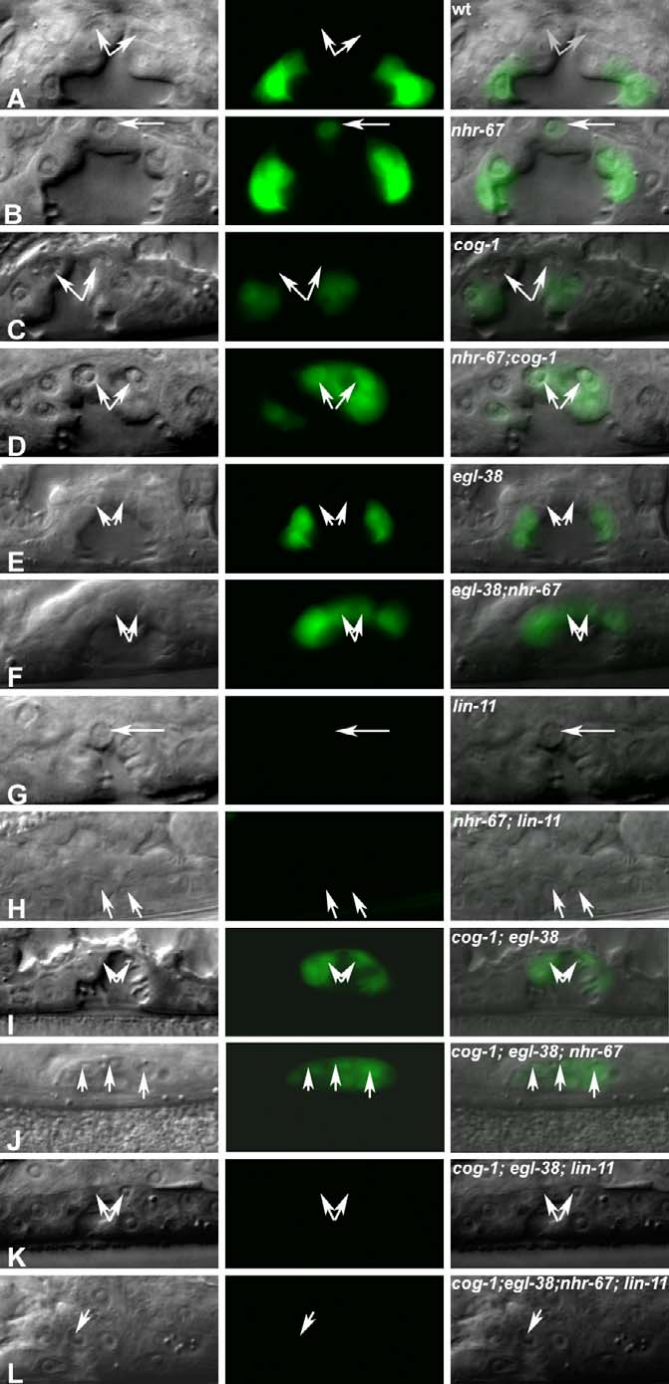
Regulation of *egl-17* Expression in the 1° Vulval Cells (A–L) Nomarski (left), fluorescence (center), and overlaid (right). Display animals from Table 1. Arrows indicate the position of the visible vulF cells during the mid L4 stage. All animals displayed carry the *ayIs4* transgene in their background. (A) In wild-type animals *egl-17* expression is absent in the vulE and vulF cells. (B) *nhr-67* RNAi typically results in ectopic *egl-17* expression in one out of four vulF cells. In contrast, *egl-17* remains off in the vulE cells. (C) In *cog-1(sy275)* mutants, *egl-17* is misexpressed in vulE cells and is absent in vulF cells. (D) *cog-1(sy275); nhr-67* RNAi doubles show a markedly stronger derepression phenotype in both 1° vulE and vulF lineages. (E) In *egl-38(n578)* mutants, *egl-17* expression is invariably off in both vulE and vulF cells. (F) *egl-38(n578); nhr-67* RNAi doubles show robust expression of *egl-17* in both vulE and vulF cells. (G) In *lin-11(n389)* animals, *egl-17* expression is absent. (H) In *lin-11(n389); nhr-67* RNAi doubles, the ectopic *egl-17* expression in vulF is eliminated. (I) *cog-1(sy275*)*; egl-38(n578)* mutants misexpress *egl-17* in vulE and vulF cells. (J) The *cog-1 (sy275*)*; egl-38(n578); nhr-67* RNAi triple shows complete *egl-17* derepression in all the vulE and vulF descendants. (K) In *cog-1(sy275*)*; egl-38(n578); lin-11(n389)* mutants, *egl-17* is completely absent. (L) In *cog-1(sy275); egl-38(n578); lin-11(n389); nhr-67* RNAi quadruples, *egl-17* expression in the vulva is abolished.

During the L4 stage, the *egl-17* transcriptional reporter is expressed solely in vulC and vulD, being absent in both vulE and vulF (Figure 5 and Table 1). *nhr-67* RNAi in an otherwise wild-type background results in an increase of *egl-17* expression in the vulF cells (Figure 5 and Table 1). In those *nhr-67* RNAi animals, only one of the four vulF cells exhibits this ectopic *egl-17* expression during the L4 stage. *egl-17* expression is consistently absent in the vulF cells of *cog-1* and *egl-38* hypomorphic alleles (Figure 5 and Table 1). In comparison, *cog-1* animals treated with *nhr-67* RNAi are qualitatively enhanced (i.e., several vulF cells misexpress *egl-17*), whereas *egl-38* animals treated with *nhr-67* RNAi displayed a qualitatively and quantitatively higher *egl-17* expression in the vulF cells (Figure 5 and Table 1). *cog-1* is necessary for negatively regulating *egl-17* expression in the vulE cells and acts redundantly with *egl-38* to negatively regulate *egl-17* in the vulF cells [19] (Figure 5 and Table 1). We also observed frequent *egl-17* upregulation in the vulE cells of *egl-38; nhr-67* (RNAi) doubly perturbed hermaphrodites (Table 1), which is invariably absent in either singly perturbed background. Our study provides the first example of *egl-38* modulating gene expression in the vulE cell type. Hence, *egl-38, nhr-67,* and *cog-1* act together to negatively regulate *egl-17* expression in the 1° vulval lineages during the L4 stage. Loss of *lin-11* function leads to complete abolition of *egl-17* gene expression in all vulval cells [26] (Figure 5 and Table 1). Lastly, the ectopic *egl-17* expression visualized in the 1° descendents of *cog-1-, egl-38-,* and *nhr-67*-depleted backgrounds is dependent on *lin-11* activity (Figure 5 and Table 1). Loss of *nhr-67* in combination with *lin-11* yields rare *egl-17* expression in apparently random vulval cell types (∼4% of animals).

**Table 1:**
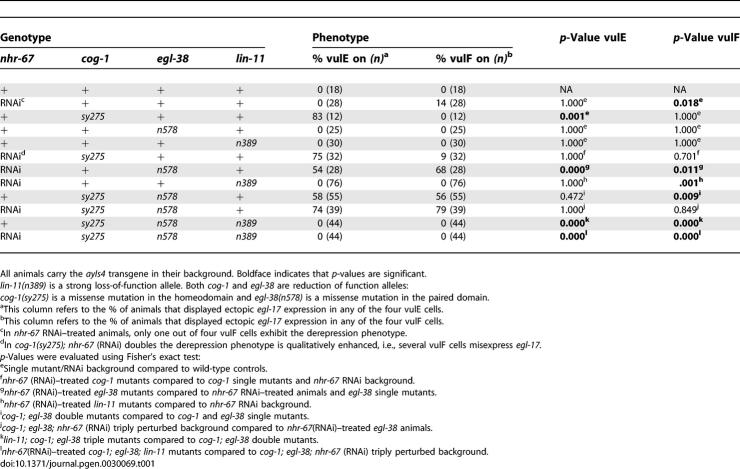
Regulation of *egl-17* Expression in the 1° Vulval Lineages

In wild-type L4 hermaphrodites, *ceh-2::YFP* expression is only observed in the vulB cells and is invariably absent in both vulE and vulF cells. *nhr-67* RNAi results in a moderate frequency of ectopic *ceh-2* expression in the vulE and vulF cells (Table 2). Eliminating *lin-11* function leads to complete loss of ectopic *ceh-2* expression in the 1° vulval lineages of *nhr-67* RNAi animals (Table 2). *ceh-2* expression is consistently absent in the 1° vulF cells of *cog-1* and *egl-38* single mutants (Table 2). *cog-1* mutants exhibit a moderate increase of *ceh-2* expression in the vulE cells [19] (Table 2). We also found that 90% of *cog-1; egl-38* doubles show increased *ceh-2* expression in vulE cells compared to *cog-1* (32%) or *egl-38* (0%) single mutants (Table 2). Thus, analysis of these double mutants provides us with a second example of *egl-38* regulating gene expression in the vulE cells. As with the *egl-17* reporter, simultaneous depletion of *cog-1* and *egl-38* activities results in a high frequency of *ceh-2* misexpression in the vulF cells (Table 2). Both *cog-1* and *egl-38* are thus required for negative regulation of *ceh-2* expression in the vulF cells.

**Table 2:**
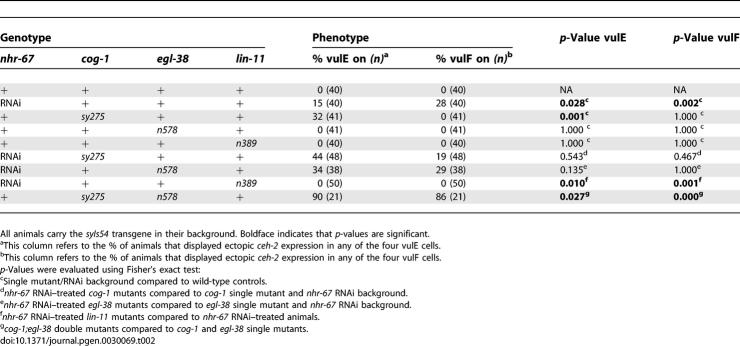
Regulation of *ceh-2* Expression in the 1° Vulval Lineages

### Regulatory Interactions between Known Components of the Vulval Patterning Network during L4

*cog-1, lin-11,* and *nhr-67*, all of which regulate different aspects of vulval gene expression, exhibit dynamic spatial and temporal expression patterns in the developing vulva [26,27]. *egl-38* expression has been observed in the vulF cells [15]. As mentioned previously, *nhr-67* expression is primarily restricted to vulA (and occasionally vulB1) cells during L4 stage. Yet numerous perturbations in gene expression are observed in *nhr-67* RNAi–treated animals, suggesting that *nhr-67* is indeed functional during the L4 stage in other mature vulval cell types besides vulA (Figure 1). A similar observation can be made about *cog-1.* Wild-type animals occasionally exhibit weak *cog-1* expression in vulE cells but none in vulF cells (Table 3). However, *cog-1* synergistically interacts with *egl-38* and *nhr-67* to regulate *egl-17* expression in the vulF cells (Figure 5 and Table 1). One attractive hypothesis is that levels of both these transcription factors are maintained under strict spatio-temporal control. We thus set out to investigate the interactions among these regulatory factors by assaying for alterations in the reporter gene expression in various mutant backgrounds.

**Table 3:**
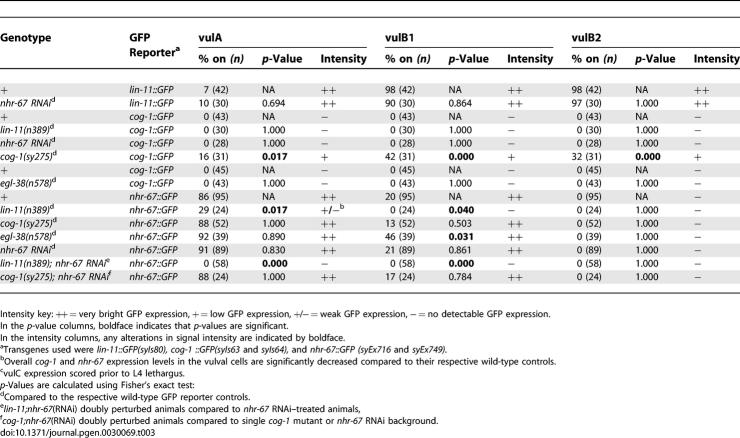
Regulatory Interactions between Known Components of the Vulval Patterning Network during L4

**Table 3:**
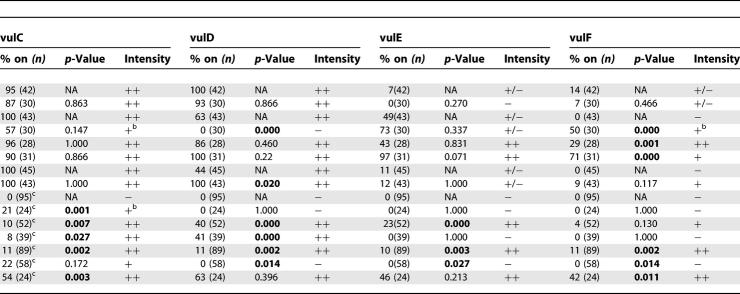
Extended.

During the L4 stage, *lin-11* is consistently expressed in the 2° vulB, vulC, and vulD lineages, and occasionally in the vulA and vulF cells. Neither *cog-1* nor *egl-38* mutations alter *lin-11* vulval expression [36]. Similarly, reduction of *nhr-67* gene activity also does not impact *lin-11* expression in the vulva (Table 3).

**Figure 6:**
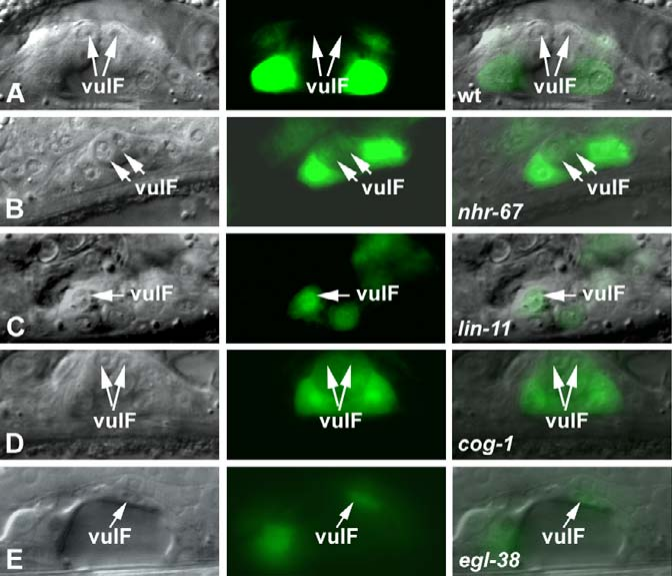
*cog-1* Levels in the 1° VulF Cells Are Antagonized by Multiple Genes (A–E) Nomarski (left), fluorescence (center), and overlaid (right). Display animals from Table 3. All animals displayed carry either the *syIs63* (A–D) or *syIs64* (E) transgene in their background. (A) In wild-type animals, *cog-1* expression is absent in the vulF cells (arrows). (B) *nhr-67* RNAi results in the derepression of *cog-1* levels in the vulF cells (arrows). (C) *lin-11(n389)* mutants show ectopic *cog-1* expression in vulF (arrow). (D) *cog-1(sy275)* mutants lose the ability to negatively autoregulate their expression levels in both vulE and vulF (arrows). (E) *cog-1* is ectopically expressed in vulF in an *egl-38* mutant background (arrow).

The *cog-1* translational reporter is strongly expressed in vulC and vulD, weakly expressed in vulE, and undetectable in vulF cells during L4 (Figure 6 and Table 3). We found that *cog-1* levels are increased in the 1° vulF cells of *nhr-67* RNAi–treated hermaphrodites as well as in *lin-11* and *egl-38* mutants (Figure 6 and Table 3). *nhr-67* RNAi–treated animals also showed elevated *cog-1* expression in the vulE cells (Table 3). In *lin-11* mutants, *cog-1* levels in vulD are completely abolished as opposed to the vulC-specific expression, which is only partially affected (∼57% of animals) (Table 3). Overall *cog-1* expression levels in *lin-11* loss-of-function mutants are noticeably reduced when compared to the wild-type reporter background. The frequency of vulD-specific *cog-1* expression is significantly increased in *egl-38* mutants (Table 3). *cog-1* negatively autoregulates in vulA, vulB1, and vulB2 cells (Table 3).

**Figure 7:**
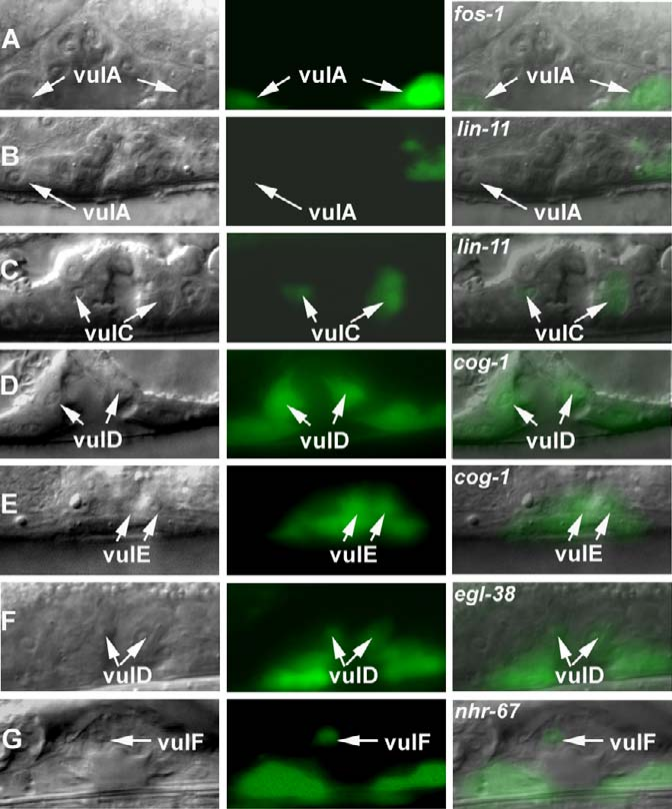
*nhr-67* Is Differentially Regulated in the 1° and 2° Vulval Lineages (A–G) Nomarski (left), fluorescence (center), and overlaid (right). Display animals from Table 3. All animals displayed carry either *syEx716* (A, D, E, and F) or *syEx749* (B and C) *[nhr-67::GFP]* transgene in their background. (A) Wild-type animals treated with control *fos-1* RNAi show no alteration of *nhr-67* vulval expression compared to non-RNAi–treated animals. *fos-1* RNAi animals exhibit abnormal vulval morphology, yet show wild-type *nhr-67* expression in vulA cells (arrows). (B) *lin-11(n389)* mutants partially eliminate the vulA-specific expression of *nhr-67* (arrow). (C) In a *lin-11(n389)* background, premature vulC expression (arrows) is observed sometimes during L4 stage. (D) *cog-1(sy275)* mutants misexpress *nhr-67* in the 2° vulD cells (arrows). (E) In a *cog-1(sy275)* background, *nhr-67* levels are highly elevated in the 1° vulE lineages (arrows). (F) *egl-38(n578)* mutants show ectopic *nhr-67* expression in vulD cells (arrows). (G) *nhr-67* RNAi feeding results in the robust increase in its own expression levels in vulF cells (arrow).

*nhr-67::GFP* expression is consistently observed in vulA during the L4 stage (Table 3). *lin-11* mutants only partially eliminate the vulA-specific expression of *nhr-67* (Figure 7 and Table 3). *nhr-67* expression in vulA is completely abolished only in the absence of both *lin-11* and its positive autoregulatory activity (Table 3). Overall, *nhr-67* expression levels in *lin-11* loss-of-function mutants are noticeably reduced when compared to a *lin-11*(*+*) background. *lin-11* activity is also required for directing the ectopic *nhr-67* expression in the 1° lineages when the autoregulatory loop is compromised (Table 3, see below). Also, loss of *lin-11* sometimes caused premature vulC expression of *nhr-67* during L4 stage, which can be interpreted either as a cell type or a temporal regulatory defect (Figure 7 and Table 3). Reduction of *cog-1* function results in increased expression of *nhr-67* in vulC and vulD during the L4 stage and vulE and vulF during L4 lethargus (Figure 7 and Table 3). Depletion of both *cog-1* and *nhr-67* activities leads to a more robust increase in *nhr-67* levels in the vulF cells (Table 3). *egl-38* mutants sometimes showed ectopic *nhr-67* expression in vulC and vulD cells during the L4 stage (Figure 7 and Table 3) and significantly increased its frequency of expression in vulB1 cells (Table 3).

### Negative Autoregulation of *nhr-67* and *cog-1* in 1° Vulval Lineages

In addition to the cross-inhibitory interactions between *cog-1* and *nhr-67* in both the 1° vulE and vulF cells, we also discovered that they both negatively autoregulate in the same cell types. Inhibition of *nhr-67* by RNAi feeding results in the robust increase of *nhr-67::GFP* expression levels in both vulE and vulF cells (Figure 7 and Table 3). Elevation of *nhr-67* transcriptional levels is also visible in the vulC and vulD lineages of *nhr-67* (RNAi) animals during L4 stage. Upregulation of *nhr-67* expression in vulC, vulD, vulE, and vulF cells is also visible with the 4.5-kb *nhr-67* transcriptional reporter construct (Figure 4B) in an *nhr-67*(RNAi) background (unpublished data). We used *fos-1* RNAi feeding as a control to exclude the possibility that the observed negative autoregulation was a nonspecific effect of inducing RNAi. *fos-1* RNAi–treated animals exhibited a strong Pvl phenotype (at least in part due to its AC invasion phenotype) [37] and did not alter *nhr-67* levels in the 1° lineages (Figure 7 and Table 3). Similarly, ectopic expression of *cog-1::GFP* in all 1° vulval descendents is consistently observed when *cog-1* activity is compromised (Figure 6 and Table 3). Thus, *nhr-67* and *cog-1* appear to be activated in all the mature vulval cell types but are then restricted by both autoregulatory and *trans-*regulatory mechanisms.

## Discussion

### *nhr-67:* A Novel Regulator of Vulval Patterning in C. elegans

*nhr-67* encodes a C. elegans ortholog of *tailless,* a crucial regulator of blastoderm patterning in the terminal pathway of *Drosophila* embryogenesis as well as neuronal development. We find that *nhr-67* activity is required for the regulation of gene expression in several mature vulval cell types and is dynamically expressed in the vulva. For technical reasons, we have been unable to determine whether *nhr-67* acts in the vulval cells for these functions. However, the expression of *nhr-67* in the vulva and the complexity of the interactions are most consistent with a primarily autonomous action of *nhr-67.* However, given the expression of *nhr-67* in the AC, it is possible that the effects (particularly on the 1° lineage) are nonautonomous. For example, the AC generates EGF and Wnt signals and is required to differentiate vulE and vulF cells, presumably via these signals [10]. Loss of vulF-specific *lin-3* expression in an *nhr-67* RNAi background is certainly consistent with this model. The AC also promotes 1° over 2° fate [38]. The ectopic expression of 2° lineage-specific genes *ceh-2* and *egl-17* in the 1° vulval cells is also consistent with this model. However, lineage analysis of *nhr-67* (RNAi) hermaphrodites argues that these alterations are not full 1° to 2° cell fate transformations in the early vulval lineages. In addition, the observed effects on *pax-2* and *zmp-1* expression are inconsistent with this model. It remains a formal possibility that some of *nhr-67* effects in the vulva are due to a role in the AC.

Our data are consistent with the function of *Drosophila tailless,* which facilitates proper gap gene expression at the posterior end of the blastoderm embryo via its dual activator/repressor activity [39–41]. Specifically, *tailless* blocks segmentation and maintains the identity of the terminal boundaries via repression of *Kruppel* and *knirps* activity and promotes *hunchback* expression, which is necessary for the establishment of terminal-specific structures [42,43]. *tailless* is also necessary for regulating gene expression during the generation of head segments as well as anterior brain development [44]. We also find that *nhr-67* prohibits improper fusion events between related cell lineages, at least partly due to strict spatial regulation of the fusogen *eff-1* in certain vulval cell types.

**Figure 8:**
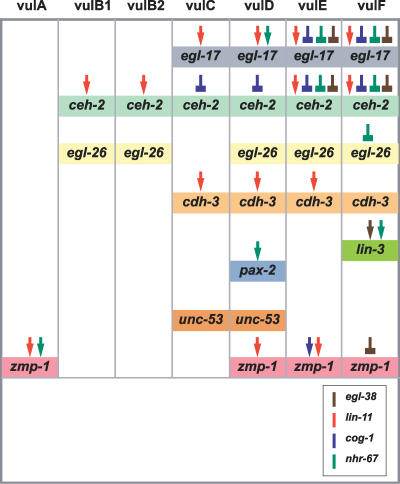
A Summary of the Gene Regulatory Network That Functions during Vulval Patterning and Differentiation in C. elegans *nhr-67* activity is included along with the other vulval patterning genes: *lin-11, cog-1, and egl-38.* Colored arrows represent positive inputs and colored block arrows represent repressor inputs for target gene expression in the distinct vulval cell types. The role of *egl-38* regulating *egl-17* and *ceh-2* gene expression in vulE is revealed by analysis of *egl-38(n578)* animals treated with *nhr-67* RNAi and *cog-1(sy275); egl-38(n578)* double mutants.

As discussed below*, nhr-67* interacts genetically with three other transcriptional regulators, *cog-1, egl-38,* and *lin-11,* to produce complex patterns of gene expression, probably through *trans*-regulation of cell type–specific enhancers (Figure 8).

### Comparison of the Vulval Network with Other Genomic Networks

We have uncovered a novel set of genetic interactions between *nhr-67,* several transcription factors, and many target genes that contribute to the identity of distinct vulval cell types. For example, *nhr-67* appears to be particularly important in the execution of vulF fate and maintaining its cellular identity via regulation of gene expression and fusion events between distinct cell types. Not only does *nhr-67* inhibit inappropriate gene expression that is associated with the 2° vulval lineages (Figure 1), but it also promotes gene expression of the EGF protein LIN-3*,* which is necessary for uv1 fate specification and facilitates proper vulval-uterine connection during development [14]. The functional data obtained from numerous RNAi experiments demonstrates that *nhr-67* (like its *Drosophila* ortholog) is a versatile regulatory gene that operates on at least four of the seven vulval cell types (vulA, vulD, vulE, and vulF). However, we have not tested whether any of these approximately ten interactions are direct.

An interesting feature of the network is our suggestion that both *nhr-67* and *cog-1* might negatively autoregulate in the same vulE and vulF cells. *Drosophila melanogaster tailless* does not regulate itself [45], suggesting that *nhr-67* autoregulation is a developmental phenomenon unique to nematodes *(C. elegans).* This apparent divergence in *tailless* regulation between phyla suggests that a more precise fine-tuning of *tailless* levels is required for the execution of accurate patterning in the C. elegans vulva. In contrast to their different autoregulatory properties, we find that certain genetic interactions are indeed conserved between the *D. melanogaster tailless* and *C. elegans nhr-67;* namely *tailless* restricts the expression domain of *ems* in the head segments [44], which is comparable to *nhr-67* repressing the worm *ems* ortholog *ceh-2* in the inappropriate vulval cells. Additional *tailless* targets from other organisms [40,46,47] may also have an impact on vulval patterning. Predictions can also be made in the reciprocal direction and used to elucidate vertebrate development. For example, FGF signaling is required for both vertebrate and inverterbrate heart development [48,49]. The LIM domain protein ISL1 promotes differentiation in a subset of cardiac progenitor cells and transcriptionally activates several FGF genes in mice [50]. Our *trans*-regulation experiments reveal that both *egl-17* and *ceh-2* contain *cis*-regulatory elements that are directly or indirectly dependent on *cog-1* (Nkx6.1/6.2), *egl-38* (Pax2/5/8), *nhr-67 (tll),* and *lin-11* (LIM) activity. These data may provide further insights into the elaborate regulation of classic developmental genes such as FGF and EMS, both of which have multiple roles in metazoan development.

### Patterning in vulE versus vulF Lineages

**Figure 9:**
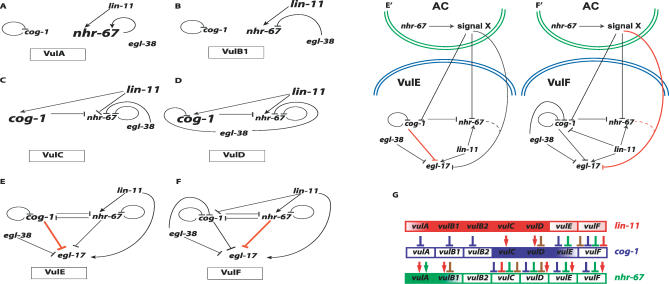
A Summary of the Identified Genetic Regulatory Interactions That Affect Some of the Distinct Vulval Cell Types In (A–F and E′ and F′), black arrows represent positive inputs and black block arrows represent repressor inputs for target gene expression that are functional within a given cell type: vulA (A), vulB (B), vulC (C), vulD (D), vulE (E and E′), and vulF (F and F′). In (A–D), the larger font size depicts high levels of expression of the represented patterning gene within the specified cell type. (E and F) This model assumes that the interactions mediated by *nhr-67* occur within the vulE and vulF cells. Red block arrows (E and F) indicate that a specific regulatory factor (*nhr-67* in vulF and *cog-1* in vulE) acts as a major repressor within the specified cell type. (E′ and F′) This alternate model presumes that *nhr-67* acts in the AC to differentiate between vulE and vulF cells. Signal X could be Ras, Wnt, or some other signaling pathway. Red block arrows (E′ and F′) indicate that the activity associated with a specific regulatory factor (*nhr-67* in vulF and *cog-1* in vulE) plays a major role in patterning gene expression with in the specified cell type. (G) A color-coded summary of the distinct expression patterns of *lin-11* (red), *cog-1* (blue), and *nhr-67* (green). Each box represents one of the seven vulval cell types. The colored boxes with the graded pattern represent rare weak expression of the transcriptional reporter, whereas boxes with solid colors represent robust expression. Colored arrows and block arrows are used to document all the identified regulatory interactions that occur during L4 patterning in the diverse vulval cell types: *lin-11* (red), *cog-1*(blue), *egl-38* (brown), and *nhr-67* (green). This model illustrates multiple patterning differences between the seven mature vulval cell types.

Previous work demonstrated that patterning of the E and F descendents of the 1° vulval lineage involves both a short-range AC-dependent signal using the Ras pathway as well as *lin-17* (Wnt) signaling [10]. In the context of *egl-17* gene expression, *cog-1* single mutants exhibit increased levels in the vulE cells only. In contrast, *nhr-67* RNAi appears to exclusively affect *egl-17* expression in the vulF cells. The negative regulatory activities of *cog-1* in vulF and *nhr-67* in vulE only become apparent in an *egl-38* mutant background (which shows no phenotype on its own). This difference suggests that *cog-1*-mediated negative regulation plays a greater role in vulE cells whereas *nhr-67-*mediated negative regulation functions primarily in vulF cells. One hypothesis is that vulF cells are biased by proximity to the AC to have higher levels of *nhr-67* compared to *cog-1* (Figure 9). The genetic regulatory interactions within the vulval network demonstrate that *cog-1* levels are negatively regulated in vulF cells via four inputs: *lin-11, egl-38, nhr-67, and cog-1.* In comparison, *nhr-67* expression in vulF cells is modulated by two antagonistic inputs *(cog-1* and *nhr-67)* and one positive input *(lin-11),* thus possibly resulting in its higher levels. These observations are consistent with a model where *nhr-67* acts as the major negative regulator in vulF cells.

*nhr-67* and *cog-1* cross-inhibit each other's transcriptional activities, specifically in the vulE and vulF cells, implying that a mutually antagonistic feedback loop exists that exclusively affects the cells of the 1° vulval lineages. Both *cog-1* and its mammalian ortholog *Nkx6.1* have been previously implicated in bistable loops that reinforce one of two possible stable end states [51,52]. The cross-inhibitory interactions between *nhr-67* and *cog-1* might be relevant in the specification of vulE versus vulF cell fates. The nature of the bistable loop between *cog-1* and *nhr-67,* however, is unknown. In particular, the bistable loop may be a consequence of either direct transcriptional regulation (as implied in Figure 9) or indirect regulation through an unknown intermediate regulatory factor.

However, the above observation does not rule out the possibility that additional regulatory factors might also contribute to proper patterning of 1° lineages. These other inputs could presumably operate via several potential mechanisms such as modulating the balance between *cog-1* versus *nhr-67* levels, being exclusively active in one 1° cell type, and interacting at distinct *cis*-regulatory elements of the downstream targets.

### Patterning Differences between 1° and 2° Vulval Lineages

Given the complexity of the observed vulval regulatory interactions, we propose that the network operating on each vulval cell type is unique (Figure 9). A single regulatory factor may have differential functions in terms of executing accurate spatio-temporal gene expression in diverse cells. For instance, *lin-11* may upregulate *cog-1* levels in the 2° vulC and vulD cells while antagonizing them in the 1° vulF cells. A similar argument can be made about the *lin-11*-dependent regulation of *nhr-67. lin-11* may temporally regulate *nhr-67* by inhibiting its vulC-specific expression during the L4 stage. In contrast, *lin-11* is clearly critical for the positive regulation of *nhr-67* expression in both vulE and vulF cells.

Both *cog-1* and *nhr-67* are present at high levels in a subset of the 2° vulval cells, yet are barely detectable in the 1° vulval cells. Nevertheless, the disruption of either factor yields obvious defects in 1° vulval cell–specific gene expression. A cross inhibition circuit, such as we propose for *cog-1* and *nhr-67,* can be bistable, with stable states that tolerate inherent fluxes in gene expression (i.e., it would not randomly oscillate between states) [53–55]. Negative autoregulatory circuits have been shown to reduce cell–cell fluctuations in the steady-state level of transcription factors [56] and can speed up the response times of transcription networks without incurring the cost of constant protein production and turnover [57]. These two distinct circuits might enable cells to reach a developmental state with built-in flexibility, allowing rapid switching of their fate upon transient inputs (as opposed to sustained inductive inputs that are metabolically costly). In this model, dynamic levels of *cog-1* and/or *nhr-67* expression could correlate with particular aspects of 1° vulval cell fate execution. This might account for the elaborate autoregulatory and *trans*-regulatory interactions specifically seen in 1° vulval descendents, as opposed to their 2°-derived counterparts. We postulate that although all the vulval cells appear to use the same regulatory factors, their differential effects on the diverse cell types is what results in accurate gene expression.

### Regulatory Code for the Seven Vulval Cell Types?

During the L4 stage, the gradient of *nhr-67* expression is opposite to that of either *cog-1* or *lin-11.* This difference in gene expression domain raises the question of whether the levels of these factors are critical for vulval development. For example, high levels of *lin-11* result in misexpression of *egl-17* in vulA and abnormal vulval invagination [26]. Different concentrations and combinatorial expression patterns of *lin-11, cog-1,* and *nhr-67* might thus encode mature vulval cell types (Figure 9). For example, differentiation to the 1° vulF cell type may entail low levels of LIN-11 and NHR-67 along with lower levels of COG-1. In contrast, the 1° vulE cells require medium levels of COG-1 along with low doses of LIN-11 and NHR-67. vulA and vulB are similar to each other with respect to maintaining low COG-1 levels. However, vulA cells are characterized by their high NHR-67 levels and medium LIN-11 levels as opposed to the reverse situation in vulB1 and vulB2 cells (medium-low NHR-67, high LIN-11). Lastly, both vulC and vulD have indistinguishably high levels of LIN-11 and COG-1, and we are unable to precisely define what distinguishes these two cell types from each other. One hypothesis is the differential regulation of NHR-67 and COG-1 in both cell types: COG-1 levels are impacted by *egl-38* in vulD (but not vulC), whereas NHR-67 levels are negatively regulated by *lin-11* in vulC (but not vulD). An obvious limitation of this proposed regulatory code is that it does not take into account other transcription factors that may potentially mediate vulval patterning.

The intricacies of vulval organogenesis can be deconstructed by rigorously elucidating the genomic networks that operate within the seven mature vulval cell types. Deciphering this regulatory code will provide valuable information on network connections and might provide insights into other examples of organogenesis.

## Materials and Methods

### Microscopy.

Transgenic worms were anesthetized using 3 mM levamisole and observed using Nomarski optics (http://www.nomarski.com). Photographs were taken with a monochrome Hamamatsu digital camera (http://www.hamamatsu.com) and Improvision Openlab 4.0.4 software (http://www.improvision.com). The fluorescent images were overlaid with their respective DIC images using Adobe photoshop 7.0.1 (http://www.adobe.com). The vulval expression patterns for all strains except *syIs49* were visualized during the late L4 stage. In the case of *syEx716,* the vulval expression was also examined during L4 lethargus and adult stage. In *syIs49* animals, vulA-specific *zmp-1::GFP* expression was scored in adults only.

### Genetics and RNAi.

C. elegans strains were cultured at 20 °C using standard protocols (Brenner, 1974). Transgenes used in this study are as follows: *syIs54 [ceh-2::GFP], syIs55 [ceh-2::YFP], syIs51 [cdh-3::CFP], syIs49 [zmp-1::GFP], syIs77 [zmp-1::YFP], syIs59 [egl-17::CFP]* [9], *syIs78 [ajm-1::GFP]* [26], *syIs107 [lin-3::GFP]* [58], *ayIs4 [egl-17::GFP]* [16], *guEx64 [pax-2::GFP]* (gift from Chamberlin lab), *kuIs36 [egl-26::GFP]* [18], *syIs63* and *syIs64* *[cog-1::GFP]* [27], *syIs80 [lin-11::GFP]* [59], *syEx716* [8-kb *nhr-67Δpes-10::GFP*], *syEx749* [8-kb *nhr-67Δpes-10::GFP*], *syEx744 [nhr-67* intron4 *Δpes-10::GFP], syEx925* [6 kb upstream *nhr-67::GFP* + 8 kb *nhr-67Δpes-10::GFP*], *syEx865* [*nhr-67p::GFP::nhr-67* int4–3′end], and *syEx756 [unc-53::GFP].* Alleles used in this study: LGI, *lin-11(n389);* LGII, *cog-1(sy275), eff-1(hy21);* LGIII, *unc-119(ed4);* LGIV, *unc-31(e169), egl-38(n578), dpy-4(e1166sd), dpy-20(e1282);* LGV, *him-5(e1490).* A complete list of strains is included in Table S2.

Transgenic lines were generated using standard microinjection protocol that produces high-copy number extrachromosomal arrays [60]. *syEx756* was generated by injecting the pNP10 construct [61] into *unc-119(ed4); him-5* background using *unc-119*(+) [62] and pBSK+ (Stratagene, http://www.stratagene.com) as coinjection markers.

A reverse genetics screen was conducted against 508 transcription factors (Table S1) from the Ahringer library (Medical Research Council Geneservice) to assay for alterations in vulval expression patterns for the *ceh-2::YFP* transgene. RNAi feeding protocol is similar to that previously described [32]. Embryos were harvested by bleaching gravid adults and were placed on a lawn of Escherichia coli strain expressing double-stranded RNA at 20 °C. Animals were scored after 36 h (during the L4 stage) using Nomarski microscopy. We resorted to *nhr-67* RNAi feeding for the rest of this study since the *nhr-67* deletion allele *(ok631)* results in L1 lethality and/or arrest (International C. elegans Knockout Consortium). All subsequent *nhr-67* RNAi feeding experiments were done as described above. *nhr-67* RNAi feeding experiments that entailed the restriction of cell fusion (via a temperature-sensitive allele of *eff-1*) were conducted at 25 °C.

### Generation of *nhr-67* reporter transgenes.

*nhr-67::Δpes-10::GFP* reporter gene constructs: The pPD97–78 vector, which includes the *Δpes-10* basal promoter driving GFP and the *unc-54* 3′ UTR (gift from Fire lab), was used as a template to generate 2-kb *Δpes-10::GFP* products. The primers used for amplification are 5′-GCTTGCATGCCTGCAGGCCTTG-3′ and 5′-AAGGGCCCGTACGGCCGACTAGTAGG-3′. All *nhr-67* gene fragments were amplified from the C08F8 cosmid and were stitched together with the *Δpes-10::GFP* fragment via PCR fusion [63] and were designated as “pdd-1 constructs.” Construct (1) consists of 1-kb promoter sequence, the entire coding region, and introns and 2 kb of the 3′ noncoding region attached to minimal *Δpes-10::GFP.* The primers used to amplify this template are 5′-CTGCTCAAAACTTTTGCTCC-3′ (forward) and 5′-CAAGGCCTGCAGGCATGCAAGCTTAAAGAACTACTGTAGTTTTTG-3′ (reverse). Construct (2) spans from the fourth intron to the 3′ noncoding region fused to minimal *Δpes-10::GFP.* This product was generated using the forward primer 5′-GTTCGATCATGGATCCTCTCC-3′ and the same reverse primer as construct (1). Construct (3) is an *nhr-67p::GFP* reporter that contains 1 kb of the native promoter stitched in-frame with a 700-bp coding fragment of GFP (amplified from the pPD95–69 vector, a gift from Fire lab). The resulting 1.7-kb gene product was subsequently fused to 4.5 kb of *nhr-67* regulatory sequences (that span from the fourth intron to the 3′ noncoding region) via PCR. Construct (4) contains 6-kb sequence upstream of the predicted first ATG of *nhr-67,* appended to minimal *Δpes-10::GFP.* The following primers were used to amplify this product: 5′-GAACCCGGCGACGTTACGGGGCTTC-3′ and 5′-CAAGGCCTGCAGGCATGCAAGCCATCTGTGAAACCGCAGTCATCAT-3′.

Reporter constructs were injected into *unc-119(ed4); him-5* worms using *unc-119*(*+*) [62] and pBSK+ (Stratagene) as coinjection markers. *lin-11(n389); syEx749* doubles were constructed by injecting the 8-kb *nhr-67::Δpes-10::GFP* construct into *lin-11(n389); unc-119(ed4); him-5* background using *unc-119*(*+*) as a rescue marker.

## Supporting Information

Figure S1 *nhr-67* RNAi Results in a Highly Penetrant Pvl and Egl Phenotype A mid-sagittal optical view of an adult *nhr-67* RNAi–treated hermaphrodite.
(5.3 MB TIF)

Figure S2 The Upstream Regulatory Sequence Drives *nhr-67* Expression in the Gonad (A and B) Nomarski (left), fluorescence (center), and overlaid (right). (A) *nhr-67* is expressed in the AC in hermaphrodites and (B) in the linker cell in males.
(3.8 MB TIF)

Table S1 List of Screened Transcription Factor RNAi Clones (51 KB XLS)

Table S2 Strain List (22 KB XLS)

### Accession Numbers

The WormBase Gene IDs (www.wormbase.org) as well as the Refseq accession numbers (www.ncbi.nlm.nih.gov/entrez/query.fcgi?db=Nucleotide) for the genes described in this study are *ajm-1:*WBGene00000100 (NM_077135; NM_077137; NM_077136; NM_171966); *cdh-3:*WBGene00000395 (NM_066286); *ceh-2:*WBGene00000429 (NM_059345); *cog-1:*WBGene00000584 (NM_182115); *eff-1:*WBGene00001159 (NM_001026819); *egl-17:*WBGene00001185( NM_075706); *egl-26:*WBGene00001193 (NM_061251); *egl-38:*WBGene00001204 (NM_069435); *lin-3:*WBGene00002992 (NM_171418;NM_171919;NM_171918); *lin-11:*WBGene00003000 (NM_060295); *nhr-67:* WBGene00003657 (NM_069693); *pax-2:*WBGene00003938 (NM_068112); *unc-53:* WBGene00006788 (NM_001027000;NM_001026999); and *zmp-1:*WBGene00006987 (NM_171138).

## Abbreviations

AC: anchor cell
EGF: epidermal growth factor
Egl: egg laying
FGF: fibroblast growth factor
GFP: green fluorescent protein
Pvl: protruding vulva
RNAi: RNA interference
