# Supplementary figures and images for: The tailless Ortholog nhr-67 Regulates Patterning of Gene Expression and Morphogenesis in the C. elegans Vulva
Source: PLoS Genet. 2007 Apr 27;3(4):e69. doi: 10.1371/journal.pgen.0030069 (PMC1857733; doi:10.1371/journal.pgen.0030069)

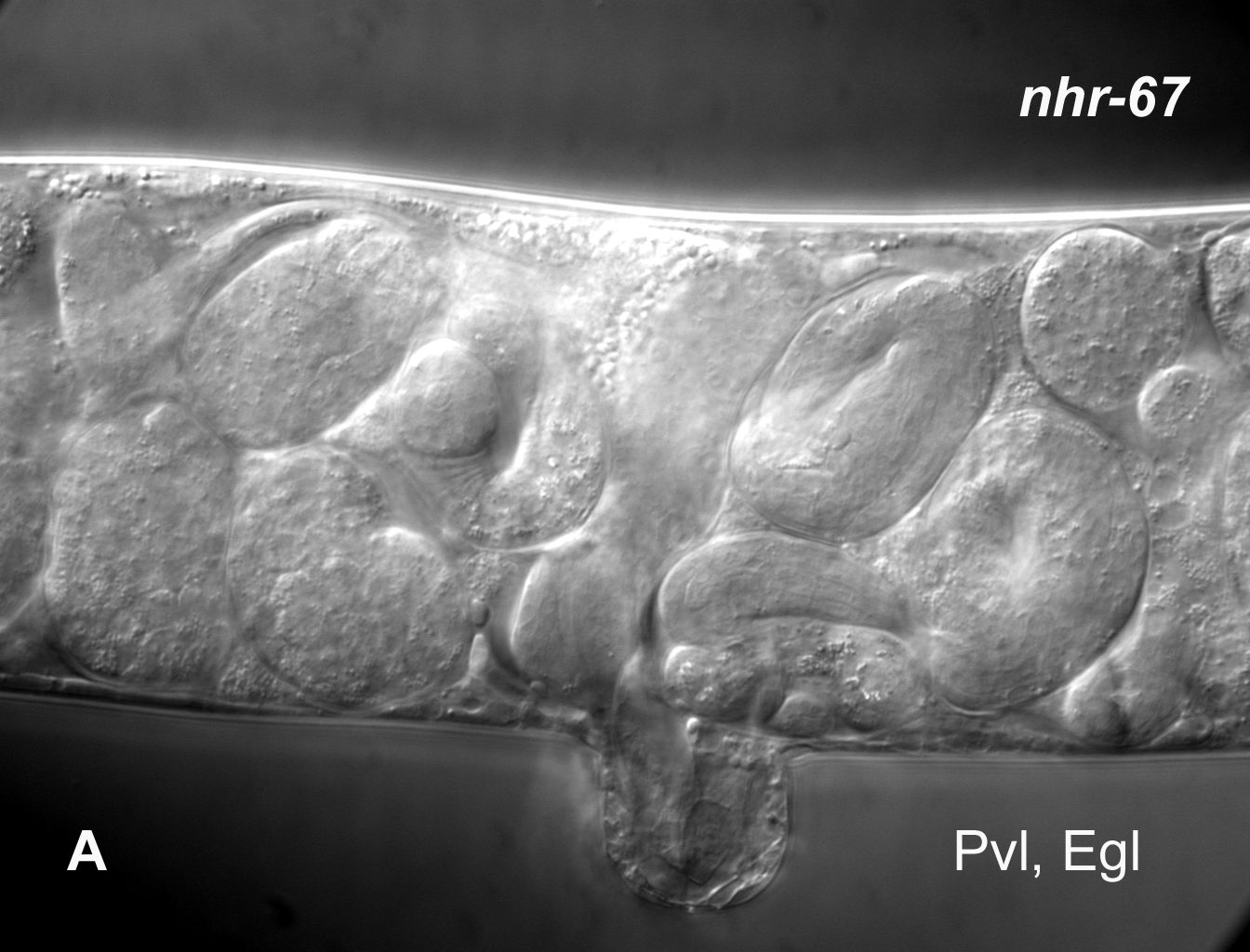

Supplement: Figure S1 — A mid-sagittal optical view of an adult nhr-67 RNAi–treated hermaphrodite. (5.3 MB TIF) [file pgen.0030069.sg001.tif]

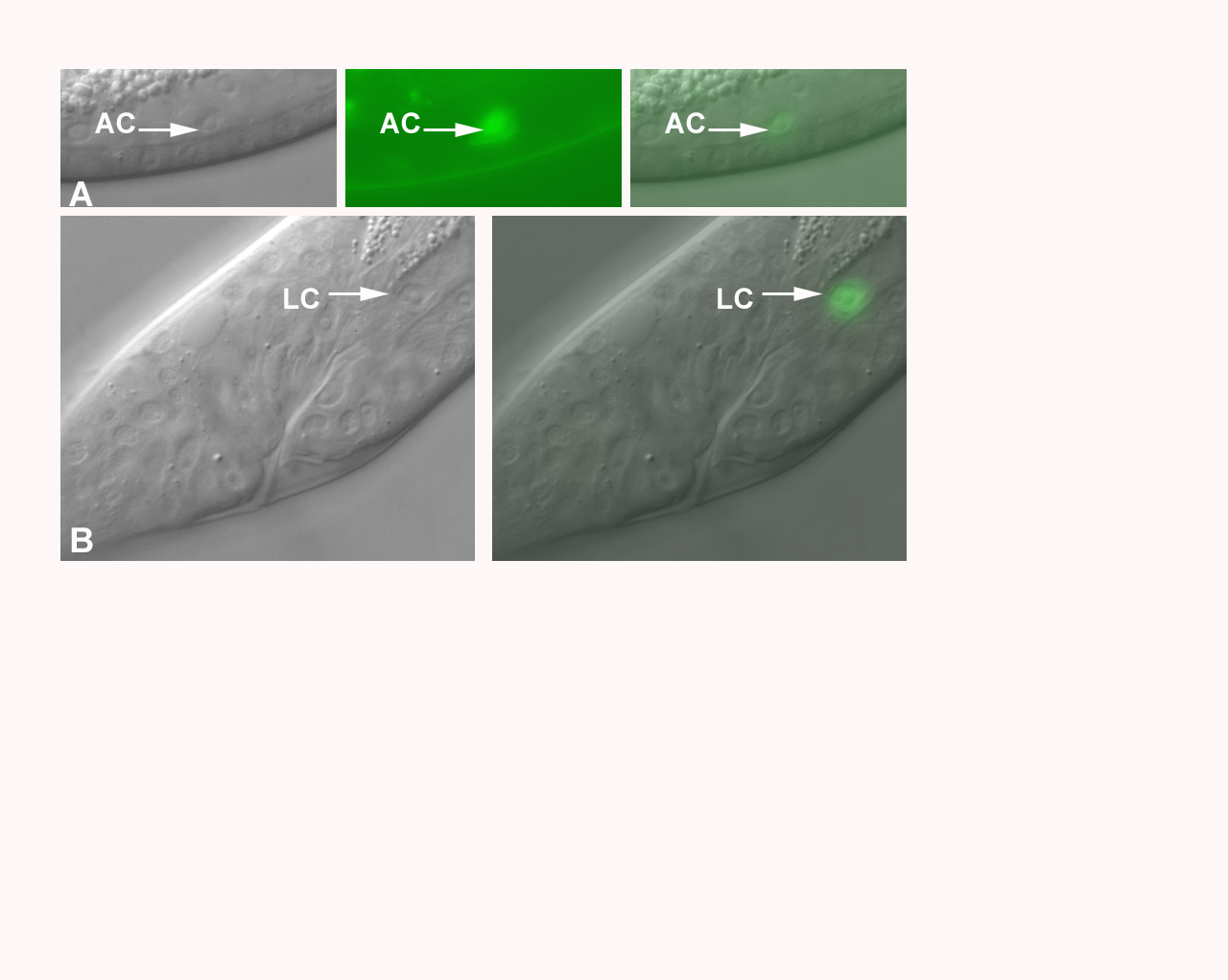

Supplement: Figure S2 — (A and B) Nomarski (left), fluorescence (center), and overlaid (right). (A) nhr-67 is expressed in the AC in hermaphrodites and (B) in the linker cell in males. (3.8 MB TIF) [file pgen.0030069.sg002.tif]
